# A Competitive Advantage of Middle-Sized Diatoms From Increasing Seawater CO_2_

**DOI:** 10.3389/fmicb.2022.838629

**Published:** 2022-05-18

**Authors:** Qi Zhang, Ya-Wei Luo

**Affiliations:** State Key Laboratory of Marine Environmental Science, College of Ocean and Earth Sciences, Xiamen University, Xiamen, China

**Keywords:** ocean acidification, diatom, CO_2_-concentrating mechanism, growth rate response, cell size, eco-physiological modeling

## Abstract

Diatoms, one of the most important phytoplankton groups, fulfill their carbon demand from seawater mainly by obtaining passively diffused carbon dioxide (CO_2_) and/or actively consuming intracellular energy to acquire bicarbonate (HCO_3_^–^). An anthropogenically induced increase in seawater CO_2_ reduces the HCO_3_^–^ requirement of diatoms, potentially saving intracellular energy and benefitting their growth. This effect is commonly speculated to be most remarkable in larger diatoms that are subject to a stronger limitation of CO_2_ supply because of their smaller surface-to-volume ratios. However, we constructed a theoretical model for diatoms and revealed a unimodal relationship between the simulated growth rate response (GRR, the ratio of growth rates under elevated and ambient CO_2_) and cell size, with the GRR peaking at a cell diameter of ∼7 μm. The simulated GRR of the smallest diatoms was low because the CO_2_ supply was nearly sufficient at the ambient level, while the decline of GRR from a cell diameter of 7 μm was simulated because the contribution of seawater CO_2_ to the total carbon demand greatly decreased and diatoms became less sensitive to CO_2_ increase. A collection of historical data in CO_2_ enrichment experiments of diatoms also showed a roughly unimodal relationship between maximal GRR and cell size. Our model further revealed that the “optimal” cell size corresponding to peak GRR enlarged with the magnitude of CO_2_ increase but diminished with elevating cellular carbon demand, leading to projection of the smallest optimal cell size in the equatorial Pacific upwelling zone. Last, we need to emphasize that the size-dependent effects of increasing CO_2_ on diatoms are multifaceted, while our model only considers the inorganic carbon supply from seawater and optimal allocation of intracellular energy. Our study proposes a competitive advantage of middle-sized diatoms and can be useful in projecting changes in the diatom community in the future acidified high-CO_2_ ocean.

## Introduction

Diatoms are one of the most important marine phytoplankton groups. They contribute 40% of primary production (e.g., Nelson et al., 1995; Tréguer and De La Rocha, 2013) and are one of the major contributors to organic carbon export in the global ocean (Buesseler, 1998; Jin et al., 2006). Marine diatoms fix carbon dioxide (CO_2_) into organic carbon through photosynthesis, which is catalyzed by ribulose-1,5-bisphosphate carboxylase/oxygenase (RuBisCO) in the Calvin cycle. However, RuBisCO has a low affinity for CO_2_, and its carboxylation is inefficient in fixing CO_2_ due to photorespiration unless its surrounding CO_2_ concentration is sufficiently high (Reiskind et al., 1989; Badger et al., 1998; Reinfelder, 2011). Meanwhile, due to the slow diffusion of CO_2_ in aqueous environments (10,000 times slower than that in air) and sluggish dehydration of bicarbonate (HCO_3_^–^) to CO_2_ (Wolf-Gladrow and Riebesell, 1997; Reinfelder, 2011), the CO_2_ concentration in contemporary surface oceans (∼10–30 μM) often limits the growth of diatoms (Reinfelder, 2011; Wu et al., 2014). Diatoms therefore use a series of processes termed the CO_2_-concentrating mechanism (CCM) to achieve high CO_2_ concentrations near RuBisCO at the cost of metabolic energy that is otherwise available for growth (Giordano et al., 2005; Raven et al., 2008; Hopkinson et al., 2011). As one of the key processes of CCM, many diatoms evolve to consume energy to acquire HCO_3_^–^, which is then catalyzed by carbonic anhydrase (CA) and converted to CO_2_ to maintain a high CO_2_ concentration near RuBisCO, leading to a high leakage of CO_2_ into seawater (Burkhardt et al., 2001; Cassar et al., 2002; Reinfelder, 2011). Some diatoms can also actively release extracellular CA (eCA) to catalyze the conversion of HCO_3_^–^ to CO_2_ in seawater (Martin and Tortell, 2008; Tortell et al., 2008; Chrachri et al., 2018).

The ocean absorbs approximately 26% of anthropogenic CO_2_ emissions (Friedlingstein et al., 2019), causing a significant increase in CO_2_ concentrations and a decrease in pH in seawater, collectively termed ocean acidification (Orr et al., 2005). It influences marine ecosystems with wide-ranging impacts, such as community structure, diversity, and carbon export (Sabine et al., 2004; Doney, 2009; Feng et al., 2009; Finkel et al., 2009; Endo et al., 2013; Mouw et al., 2016; Deppeler et al., 2018; Doney et al., 2020). The increasing CO_2_ concentration in surface seawater, predicted to double within this century under business-as-usual scenarios (Lerman et al., 2011), will mitigate CO_2_ limitation on phytoplankton and downregulate CCM to save intracellular energy and potentially enhance cell growth (Taylor et al., 2012; McMinn et al., 2014; Cyronak et al., 2016; Biswas et al., 2017; Gafar et al., 2018). This stimulation has been observed in CO_2_ enrichment experiments of diatoms when they were cultured under favorable nutrient and light conditions (e.g., Kroeker et al., 2013; Wu et al., 2014).

Diatoms vary greatly in cell size, from a minimal reported dimension < 1 μm to a maximal reported dimension of >5,000 μm in a global marine diatom database (Leblanc et al., 2012a,b). However, large diatoms are usually less abundant in the community, mainly because of their decreasing growth rates with cell size (Mizuno, 1991; Sarthou et al., 2005). For example, in 515 diatom species sampled along the Swedish coast and the Gulf of Finland, those with a cell volume larger than 1,000 μm^3^, an equivalent spherical diameter equivalent (ESD) of 12 μm, accounted for only approximately half (57%) of the total species (Snoeijs et al., 2002). In this study, we followed that study and define the “large” diatoms by ESD of larger than 12 μm. We further defined the “small” and “middle-sized” diatoms by ESD of < 5 μm and 5–12 μm, respectively.

The relationship between diatom cell size and the degree of their growth rate response (GRR), defined here as the ratio of the growth rates under elevated and ambient CO_2_ concentrations, remains unclear. If CO_2_ was the only carbon source for diatoms, their GRR would increase with cell size because larger cells have smaller surface-to-volume ratios and are limited more severely by CO_2_ supply (Wirtz, 2011). However, HCO_3_^–^ is the secondary carbon source of diatoms, and its concentration in seawater (∼10^3^ μM) is 2 orders of magnitude higher than that of CO_2_. It is worth further evaluating how diatoms respond differently to increasing CO_2_ when they also acquire substantial amounts of HCO_3_^–^.

Although the increase in GRR with cell size has been shown in a CO_2_ enrichment experiment of diatoms (Wu et al., 2014), the interpretation of the results can be complex, such as the high light intensity of 350 μmol photons/m^2^/s used in that experiment. Indeed, high light can inhibit the photosynthesis of diatoms, which can be exacerbated when cells grow in acidified, high-CO_2_ environments. For example, the effect of increasing CO_2_ on the growth of three diatom species shifted from positive to negative when the incubation light increased from 150 to 250 μmol photons/m^2^/s (Gao et al., 2012). It is therefore possible that the higher GRR seen in larger diatoms in Wu et al. (2014) can be partly a result that larger cells were less susceptible to the inhibition of high light (Key et al., 2010) instead of a sole effect of mitigated CO_2_ limitation.

In this study, we constructed a theoretical model to simulate the GRR as a function of cell size and magnitude of seawater CO_2_ increase. The model calculated the CO_2_ supply rate to diatom cells as a result of physical diffusion and carbonate equilibrium in seawater, represented CO_2_ leakage from cells, and solved an optimal energy allocation between HCO_3_^–^ acquisition and biomass synthesis. The model results were compared to historical data from diatom CO_2_ enrichment experiments. We also evaluated the robustness of our model and further explored the underlying mechanisms by performing a series of model sensitivity experiments. We finally applied our model to the global ocean under a CO_2_ release scenario (Representative Concentration Pathway RCP 8.5), illuminating possible spatial variations in the cell size–dependent responses of diatom growth.

## Materials and Methods

### Model Overview

**FIGURE 1:**
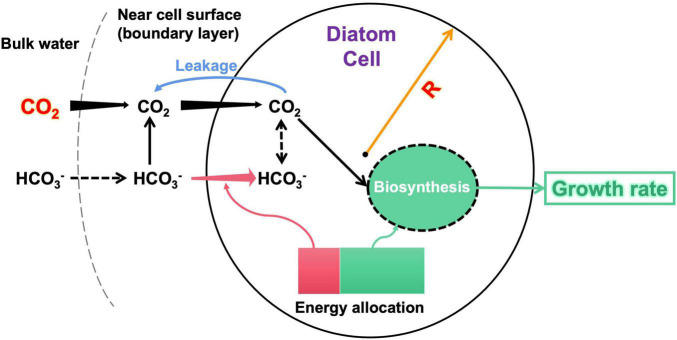
Illumination of the model structure. The input variables of the model are diatom cell radius R and bulk-water CO_2_ concentration. The model estimates the CO_2_ supply (black arrows) to the cell considering both CO_2_ diffusion and HCO_3_^–^ dehydration near the cell surface, energy-consuming HCO_3_^–^ transport (pink arrows), and CO_2_ leakage (blue arrow) across cell membranes. The model finally calculates an optimal allocation of metabolic energy between HCO_3_^–^ acquisition and biomass synthesis, which derives the growth rate as the model output. An arrow with increasing (decreasing) width along the direction represents that the flux increases (decreases) with the cell radius. The dashed arrows are not explicitly simulated by the model. More details of the model are described in the text.

We constructed a model of a spherical diatom cell to quantify its acquisition rates of CO_2_ and/or HCO_3_^–^ and to solve for the optimal allocation of metabolic energy between HCO_3_^–^ uptake and biomass synthesis (i.e., growth) over a day (Figure 1). Given cell radius *R* (μm) and CO_2_ concentration in bulk water [CO_2_]_bulk_ (μM), the model simulates a daily growth rate *g* /day:


(1) $$\text{g}=\text{f}\,\left(R,{\left[{\mathrm{CO}}_{2}\right]}_{\mathrm{bulk}}\right)$$

Note that although the model uses the cell radius to simplify calculations, the results are conventionally reported using the cell diameter.

The model first calculates the cell-specific acquisition rate of CO_2_ under given *R* and [CO_2_]_bulk_. The model then counted the CO_2_ leakage from the cell. The model reaches a maximal growth rate if the net acquisition of CO_2_ is sufficient; otherwise, it lowers the growth rate to save metabolic energy for actively acquiring HCO_3_^–^ as a carbon complement. The lowered growth also reduces the total carbon demand, partly relieving the requirement for HCO_3_^–^. Note that we set a minimum HCO_3_^–^ acquisition at 20% of the total carbon demand even if the CO_2_ supply is sufficient, reflecting findings that even very small diatoms also take HCO_3_^–^ (e.g., Burkhardt et al., 2001; Shi et al., 2019). Therefore, there exists a solution for the modeled growth rate at which cellular energy is optimally allocated between HCO_3_^–^ acquisition and growth.

The parameter values of the model (Supplementary Table 1) were set up based on the literature as described below. Our model only represents fundamental metabolic processes of cell growth and focuses on the responses of diatom growth to increasing CO_2_, particularly the dynamics of HCO_3_^–^ acquisition as the secondary inorganic carbon source, while ignoring other complex physiological processes.

### Model Scheme of the CO_2_ Supply

Our model scheme of the CO_2_ supply rate to the cell (*F_T_*, μmol/cell/day) is established by improving those of previous studies (Gavis and Ferguson, 1975; Wolf-Gladrow and Riebesell, 1997; Reinfelder, 2011) in which both CO_2_ diffusion and CO_2_ dehydrated from HCO_3_^–^ near the cell surface are included


$${\text{F}}_{T}=3.456\times 10^{-10}\cdot \pi \,\text{DR}\,\left(1+\text{R}\,\sqrt{\frac{{\text{k}}^{{}^{\prime }}}{\text{D}}}\right)$$

(2) $$\,\left({\left[{\text{CO}}_{2}\right]}_{\mathrm{bulk}}-{\left[{\text{CO}}_{2}\right]}_{\mathrm{sfc}}\right)$$

where 3.456 × 10^–10^ = 4 × 10^–15^ L ⋅μm^–3^ × 86,400 s/day, in which the constant of 4 is from the original equation (Gavis and Ferguson, 1975), 10^–15^ is the factor to convert μm^3^ to L for consistency with the unit used in CO_2_ concentrations, and 86,400 is the factor to convert day to second; *D* (μm^2^/s) is the diffusivity of CO_2_ in water *k*′ (s^–1^) is a combined rate constant for the hydration of CO_2_ with H_2_O and OH^–^, with its value estimated at 15°C, pH of 8.1 and salinity of 35 PSU (Supplementary Table 1; Wolf-Gladrow and Riebesell, 1997; Reinfelder, 2011), and [CO_2_]_sfc_ (μM = μmol L^–1^) is the seawater CO_2_ concentration near the cell surface. Although [CO_2_]_sfc_ can be effectively eliminated from the equation by assuming that it was 1/3 of [CO_2_]_bulk_, as done in a previous study (Reinfelder, 2011), [CO_2_]_sfc_ is likely to decline with increasing cell size (Flynn et al., 2012). In this study, we further solve [CO_2_]_sfc_ by assuming a balance between CO_2_ supply to the cell surface (*F_T_*) and CO_2_ permeation across the cell membrane (*F_P_*, μmol/cell/day):


(3) $${\text{F}}_{T}={\text{F}}_{P}=3.456\times 10^{-10}\cdot \pi \,{\text{R}}^{2}\,\text{P}\,\Delta_{\mathrm{CO2}}\,{\left[{\text{CO}}_{2}\right]}_{\mathrm{sfc}}$$

where *P* (μm/s) is the CO_2_ permeability coefficient of the cell membrane, Δ_*CO2*_ = 10% is the relative reduction in CO_2_ concentration from near the cell surface to the cytoplasm (Hopkinson et al., 2016), that is, Δ_*CO2*_[CO_2_]_sfc_ is the difference in CO_2_ concentration between the two sides of the cell membrane, and 3.456 × 10^–10^ = 4 × 10^–15^ L ⋅μm^–3^ × 86,400 s/day. From Eqs. 2 and 3, [CO_2_]_sfc_ can be solved as a function of input variables *R* and[CO_2_]_bulk_:


(4) $${\left[{\mathrm{CO}}_{2}\right]}_{\mathrm{sfc}}=\frac{{\text{D}}^{{}^{\prime }}\,{\left[{\mathrm{CO}}_{2}\right]}_{\mathrm{bulk}}}{\text{RP}\,\Delta_{\mathrm{CO2}}+{\text{D}}^{{}^{\prime }}}$$

where $D^{\prime }=D\cdot \left(1+R\,\sqrt{k^{\prime }/D}\right)$. This equation generates a declining [CO_2_]_sfc_ with increasing cell size, as expected (Supplementary Figure 1). *F_T_* can then be obtained by substituting Eq. 2 into either Eq. 3 or Eq. 4.

This scheme introduces a new parameter *P*, for which the maximal value of 560 μm/s found in experiments of several diatom species (Hopkinson et al., 2011) is used in the model (Supplementary Table 1). The assumption of *F*_*T*_=*F*_*P*_ used in the scheme (Eq. 3) implies that CO_2_ supplied to the cell surface is completely acquired by the cell, and the CO_2_ supply rate is solved at its maximal potential, which we annotate as *F*_*T,max*_.

### Model Scheme of Energy Constraint

The energetic cost of growth is estimated by adding the theoretical requirements of ATP when synthesizing particulate organic carbon (POC) from CO_2_ [3 mol ATP (mol C) ^–1^] (Raven, 1991) and synthesizing particulate organic nitrogen (PON) from nitrate [2 mol ATP (mol N) ^–1^] (Eichner et al., 2014), which is equivalent to ∼ 0.30 ATP (mol C) ^–1^ by using a Redfieldian molar C:N ratio of 106:16. By further assuming small additional energy of 0.2 mol ATP (mol C) ^–1^ used in other cellular processes, the energy cost rate for growth, *e_g_*, is set at 3.5 ATP (mol C) ^–1^ (Supplementary Table 1).

The intracellular energy production rate *E_0_* [ATP/mol C/day] is set at:


(5) $${\text{E}}_{0}={\text{g}}_{0}\cdot {\text{e}}_{g}$$

where the maximal daily average growth rate *g_0_* = 3.0/day represents those maximal rates found in previous experiments culturing diatoms under saturating light and nutrients (Sarthou et al., 2005). The instantaneous maximal growth rate (and consequently, *E_0_*) is further set to vary in a 12:12 h light–dark cycle in which the rate peaks for 6 h around noon to represent light saturation, while its daily average is kept at *g_0_* (Supplementary Figure 2). This setup can improve the model performance to mimic the diel variation of inorganic carbon demand. For example, a model cell that would have sufficient CO_2_ supply, should its maximal growth rate be set constant at *g_0_*, may become, after implementing the diel cycle, CO_2_-limiting and have to acquire HCO_3_^–^ around noon when the instantaneous maximal growth rate is 8/3 times higher than *g_0_* (Supplementary Figure 2).

ATP expenditure by CCM is generally high in diatoms (Raven, 1991), which may be partially satisfied by the Mehler reaction (Behrenfeld et al., 2008). The energy cost for diatoms to transport 1 HCO_3_^–^ molecule has been estimated at 0.5 ATP to cross the cytoplasmic membrane (Liu et al., 2017) and 1 ATP to cross the chloroplast membrane to reach pyrenoids (Raven et al., 2000). Therefore, we set the energy cost rate for HCO_3_^–^ acquisition, *e*_*bc*_, at 1.5 ATP (mol C) ^–1^ (Supplementary Table 1).

### Model Scheme of Growth Rate

The net inorganic carbon acquisition rate *F* (μmol/cell/day) is


(6) $$\text{F}=\left({\text{F}}_{T}+{\text{F}}_{\mathrm{bc}}\right)\cdot \left(1-{\text{l}}_{k}\right)$$

where *F*_*bc*_ (μmol/cell/day) is the HCO_3_^–^ acquisition rate, and *l_k_* = 30% is the fraction of acquired carbon leaking from the cell based on a previously reported CO_2_ efflux for diatoms (Burkhardt et al., 2001). Note that the leakage in the model does not count the exudation, which, however, could be small (∼5% of primary production) in exponentially growing phytoplankton (Nagata, 2000). The model sensitivity to *l_k_* was also tested (see below).

If both *F_T_* and *F*_*bc*_ are determined (discussed below), *F* can be solved (Eq. 6), and the growth rate is then


(7) $$\text{g}=\text{F}/{\text{q}}_{c}$$

where *q_c_* is the cell carbon quota (μmol cell^–1^) estimated by adopting an empirical relationship with the cell volume of diatoms (Menden-Deuer and Lessard, 2000):


(8) $${\text{q}}_{c}=2.4\times 10^{-8}\cdot {\left(\frac{4}{3}\,\pi \,{\text{R}}^{3}\right)}^{0.811}$$

*F_T_* and *F*_*bc*_ are optimally solved under the constraint of intracellular energy production *E_0_*. The intracellular energy requirement is:


(9) $$\text{E}={\text{E}}_{g}+{\text{E}}_{\mathrm{bc}}$$

in which


(10) $${\text{E}}_{g}={\text{e}}_{g}\cdot \text{g}={\text{e}}_{g}\cdot \left({\text{F}}_{T}+{\text{F}}_{\mathrm{bc}}\right)\cdot \left(1-{\text{l}}_{k}\right)/{\text{q}}_{c}$$

(11) $${\text{E}}_{\mathrm{bc}}={\text{e}}_{\mathrm{bc}}\cdot {\text{F}}_{\mathrm{bc}}/{\text{q}}_{c}$$

A trial rate of HCO_3_^–^ acquisition (*F*_*bc*_) is first set to be the minimal fraction ($f_{m\,i\,n}^{b\,c}$) of total carbon uptake (*F*_*T,max*_ + *F*_*bc*_) so that:


(12) $${\text{F}}_{\mathrm{bc}}={\text{F}}_{T,\max}\cdot {\text{f}}_{\min}^{\mathrm{bc}}/\left(1-{\text{f}}_{\min}^{\mathrm{bc}}\right)$$

Then, a trial energy requirement *E* is calculated using Eqs. 10–12 and setting *F_T_* = *F*_*T,max*_ (see section “Model Scheme of the CO_2_ Supply”). When *E* > *E_0_*, energy production limits growth and the total carbon supply is more than sufficient. Therefore, the effective *F_T_* and *F*_*bc*_ must be smaller than their trial rates while their ratio holds. Therefore, a factor of *E_0_*/*E* is multiplied by *F*_*T,max*_ and the trial *F*_*bc*_ to obtain the effective *F_T_* and *F*_*bc*_, which ensures *E* = *E_0_*.

However, a trial rate of *E* larger than *E_0_* indicates that the inorganic carbon supply is insufficient, additional acquisition of HCO_3_^–^ is needed, and the effective *F_T_* is at its maximal allowed rate *F*_*T,max*_. The effective *F*_*bc*_ is calculated from Eqs. 9–11 by setting *E* = *E_0_* (Eq. 9) and *F_T_* = *F*_*T,max*_ (Eq. 10).

Once the effective *F_T_* and *F*_*bc*_ are determined in either case, the growth rate *g* can be calculated from Eqs. 6, 7. Noting that the intracellular energy produced is fully used, the scheme described here solves an optimal allocation of intracellular energy to estimate *g* at its maximal potential.

### Model Simulation

The cell growth rate was simulated at two levels of bulk-water CO_2_ concentrations of 10 μM (low carbon condition, LC) and 20 μM (high carbon condition, HC) over a diameter range of 2–200 μm. LC was selected at 10 μM to represent the typical condition of subtropical surface ocean waters. GRR is calculated as the ratio of the simulated growth rates under HC and LC. We also calculated two key indicators, the value of the peak GRR over the simulated diameter range and the corresponding cell diameter at the peak GRR (namely, the optimal cell diameter).

### Sensitivity Tests

Our model does not represent other factors, such as light and nutrient concentrations, that can limit the diatom growth rate. Instead, the model parameter *g_0_* can be considered to represent collective effects from multiple limiting factors. We then conducted model experiments by setting *g_0_* at 0.1, 0.5, 1.0, and 2.0/day.

We also compared the model sensitivity, in terms of the peak GRR and the optimal cell diameter, to 6 model parameters, including *P*, *l_k_*, *e*_*bc*_, *g_0_*, △_*CO2*_, and $f_{m\,i\,n}^{b\,c}$. These parameters were tested in a range of ± 50% of their default values. The model was also tested over 12–30 μM of the input variable [CO_2_]_bulk_ in HC.

### Analysis of Historical Data of Diatom CO_2_ Enrichment Experiments

We collected 85 pairs of growth rate data from published CO_2_ enrichment experiments culturing diatoms, among which 5 pairs of data were removed due to extremely high culturing CO_2_ concentrations (Supplementary Table 2). Due to different magnitudes of CO_2_ enrichment, we linearly adjusted the originally reported GRR (GRR_orig_) cultured under high ([CO_2_]_H_) and low ([CO_2_]_L_) CO_2_ concentrations to GRR_adj_ at a CO_2_-enrichment factor (*f*_*CO2*_) of 200%:


(13) $$\frac{{\text{GRR}}_{\mathrm{adj}}-100\%}{{\text{f}}_{\mathrm{CO2}}-100\%}=\frac{{\text{GRR}}_{\mathrm{orig}}-100\%}{{\left[{\text{CO}}_{2}\right]}_{H}/{\left[{\text{CO}}_{2}\right]}_{L}-100\%}$$

The cell diameters of the diatoms were either reported in the experiments or estimated using the average diameters of the same species collected in a global diatom database (Leblanc et al., 2012a). Considering the purpose of this study, for nonspherical diatom species, we simply used the length of their shortest dimension as the cell diameter because it was the shortest distance that inorganic carbon was transported to the cell center, admitting that this simplification slightly overestimated the CO_2_ supply rate.

### Global Prediction

We projected the peak GRR and optimal cell size of diatoms to CO_2_ increase in global surface seawater over the 21st century. We first obtained projected results from the Community Earth System Model (CESM) 1.0-BGC module under the RCP8.5 scenario, including the annual climatology of CO_2_ partial pressure, pH, temperature, diatom carbon fixation rate, and diatom carbon biomass during the historical (1990–2000) and future (2090–2100) periods^1^. The CO_2_ partial pressure, pH, and temperature were used to calculate the CO_2_ concentration using CO2SYS (Lewis and Wallace, 1998). The diatom growth rates were calculated as the ratio of the diatom carbon fixation rate to the diatom carbon biomass.

The cellular model ran using historical and future CO_2_ concentrations to estimate monthly peak GRR and optimal cell size at a resolution of 1°× 1° in the global ocean, in which historical diatom growth rates were used to set location specificity *g_0_*. Because CESM did not simulate diatom sizes, our results can only be regarded as a demonstration of the spatial variations in the responses of diatoms impacted by the magnitude of CO_2_ increase and environmental favorability (such as nutrients, temperature, and light), with the latter collectively represented here by the CESM-projected diatom growth rate.

The scientific color map batlow was used in the plot of the global prediction to prevent visual distortion of the data and exclusion of readers with color-vision deficiencies (Crameri et al., 2020).

## Results

### Model Results

**FIGURE 2:**
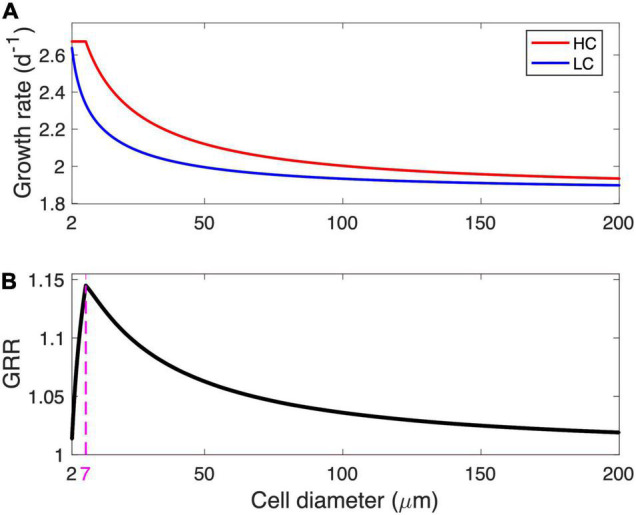
Modeled growth rate and growth rate response (GRR). **(A)** Modeled growth rate under low-CO_2_ (LC, 10 μM) and high-CO_2_ (HC, 20 μM) conditions and **(B)** the corresponding GRR from LC to HC as a function of cell diameter. The magenta dashed line and number in **(B)** represent the optimal cell diameter at peak GRR.

The modeled cell growth rate declines with increasing cell diameter under LC, while the modeled growth under HC remains at a maximal rate of 2.67/day before it starts to decline when its diameter is larger than 7 μm (Figure 2A). As the ratio of the modeled growth rate under HC to that under LC, the GRR increases with cell diameter, reaches a peak value of 1.14 at an optimal cell diameter of 7 μm, and then gradually decreases to 1.02 at the modeled maximal cell diameter (Figure 2B).

**FIGURE 3:**
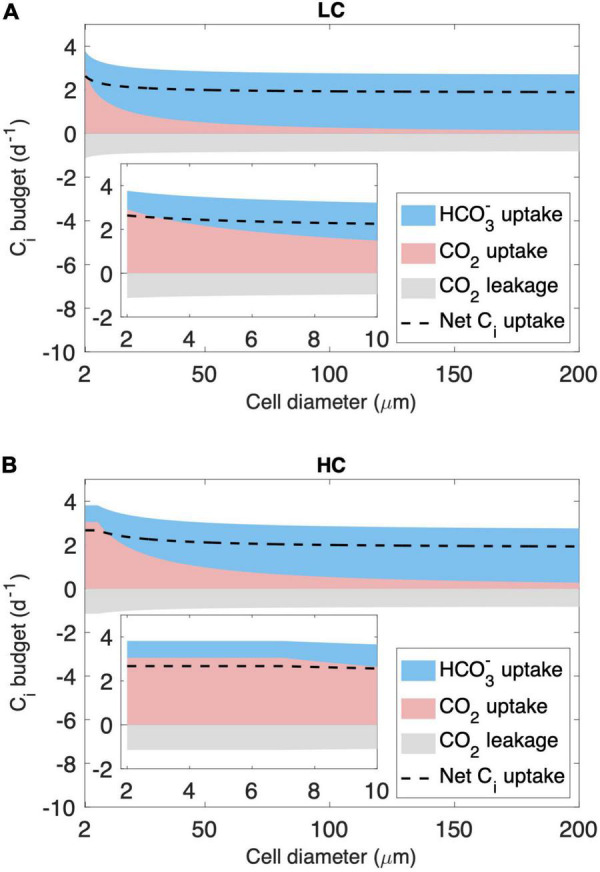
Modeled inorganic carbon (Ci) budget (normalized to cell carbon biomass) across the cytoplasmic membrane. The results shown include those under **(A)** the low-CO_2_ (LC, 10 μM) and **(B)** high-CO_2_ (HC, 20 μM) conditions in the cell diameter of the full modeled range and zoomed in to 2–10 μm. Ci is acquired in the form of HCO_3_^–^ (blue area) and CO_2_ (pink area), and part of the CO_2_ leaks out of the cell (gray area), resulting in a net Ci uptake used for cell growth (dashed line).

We then analyze the inorganic carbon (C_*i*_) budget of the model cell in two cell diameter ranges. As expected, the model simulates a decreasing CO_2_ supply rate with increasing cell diameter (Figure 3). First, for cells with a diameter < 7 μm, CO_2_ and minimum HCO_3_^–^ supply under LC are insufficient to meet the requirements of both growth and leakage, causing HCO_3_^–^ acquisition to increase with cell size (Figure 3A). However, under HC, CO_2_ and minimum HCO_3_^–^ supply are sufficient, and cells do not acquire additional HCO_3_^–^ (Figure 3B). Second, for cells with a diameter > 7 μm, CO_2_ and minimum HCO_3_^–^ supply become insufficient even under HC (Figure 3B), and the modeled HCO_3_^–^ acquisition increases with cell size under both HC and LC (Figure 3).

**FIGURE 4:**
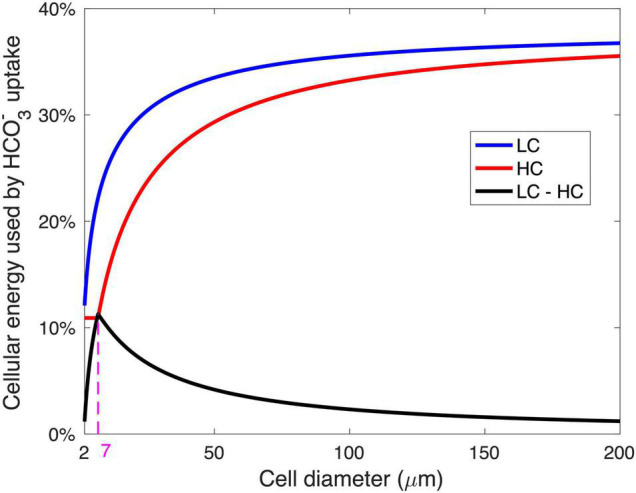
The relationship between the modeled energy consumption for HCO_3_^–^ acquisition and the cell diameter. The energy for HCO_3_^–^ acquisition is shown as the fraction of total cellular metabolic energy under the low-CO_2_ (LC, 10 μM) (blue) and high-CO_2_ (HC, 20 μM) (red) conditions, as well as the saved energy in HCO_3_^–^ acquisition when the condition changed from LC to HC (black). The magenta dashed line and number represent the optimal cell diameter at maximal energy savings from LC and HC.

The modeled pattern between GRR and cell size (Figure 2B) is mainly determined by the magnitude of energy savings on HCO_3_^–^ acquisition from LC to HC (Figure 4). In the cell diameter range < 7 μm, the energy expenditure on HCO_3_^–^ increases greatly with increasing cell size under LC but is unchanged under HC, resulting in substantial energy savings that increase with cell size (Figure 4). In the cell diameter range > 7 μm, however, the energy used in HCO_3_^–^ acquisition increases with cell size under both HC and LC, leading to a narrowed difference between the two (Figure 4). This can be explained by the decrease in the relative contribution of CO_2_ to the total C_*i*_ acquisition with increasing cell size (Figure 3): The fraction of HCO_3_^–^ acquisition that can be substituted by the same magnitude of the elevated CO_2_ supply decreases in larger diatoms. In other words, larger cells become less sensitive to the CO_2_ increase.

The model reveals a general pattern in which (1) the smallest diatoms respond weakly to the increase in seawater CO_2_ because CO_2_ supply fulfills most of the cell demand even without that increase, (2) the response intensifies with a moderate enlargement in cell size, while (3) large cells are insensitive to the CO_2_ increase because CO_2_ supply is a small contribution to total C_*i*_ acquisition. There exists an optimal, intermediate cell size in which the modeled cell growth rate is enhanced the most by the same magnitude of CO_2_ increase. Our model shows that the optimal cell size corresponds to a point where the model cell under HC just starts to use HCO_3_^–^.

### Sensitivity Tests

**FIGURE 5:**
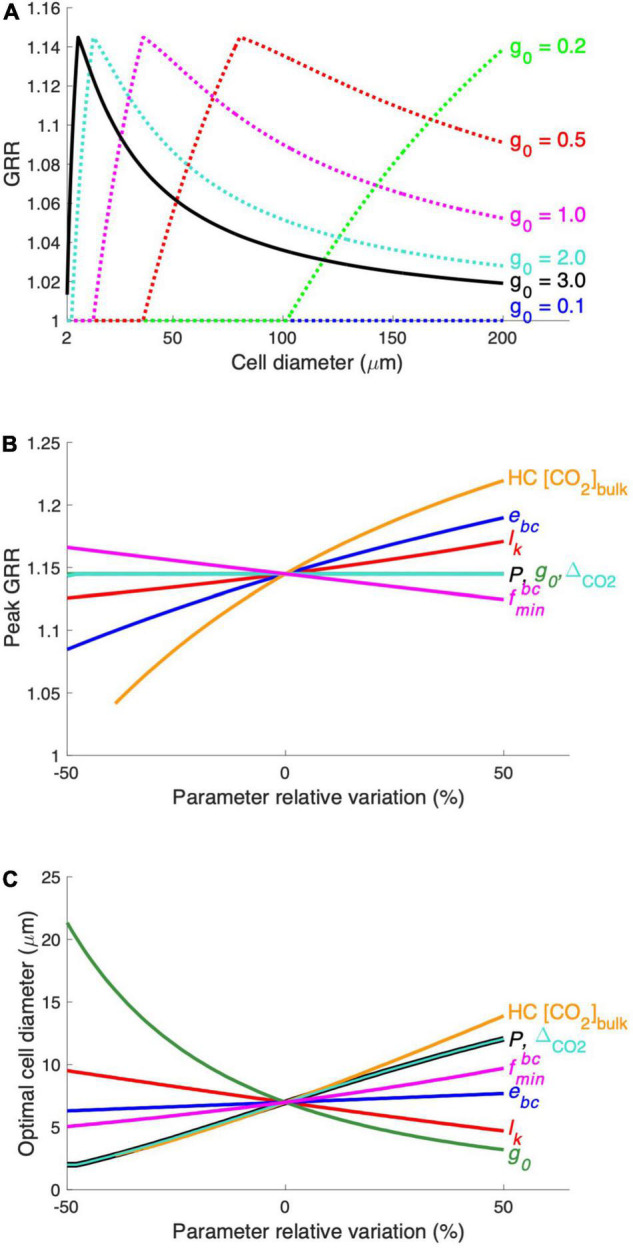
Results of the parameter sensitivity experiment. **(A)** The modeled relationship between GRR and cell diameter is tested at different levels of the model parameter *g_0_* (maximal growth rate). The modeled peak GRR **(B)** and the optimal cell diameter **(C)** were tested against ± 50% of the default values of the parameters. See Supplementary Table 1 for parameter symbols and their default values. The range of HC CO_2_ concentrations (HC [CO_2_]_bulk_) tested in this experiment was 12–30 μM.

The maximal growth rate (*g_0_*) has been set at a high level assuming that the modeled diatom grows at optimal conditions. We then conduct the model experiments with lowered *g_0_*, representing that the growth of the modeled diatom is limited by other factors. The results show that the modeled diatom starts to benefit from the CO_2_ increase at larger cell sizes when *g_0_* decreases, so that the optimal cell size also increases (Figure 5A). This result is expected because the modeled diatom needs less inorganic carbon at a lowered *g_0_*, and the CO_2_ supply alone becomes sufficient for larger diatoms. At very low *g_0_* values, such as 0.1/day, the CO_2_ supply is even sufficient at the maximal cell diameter simulated in our model (200 μm), and diatoms do not respond to the CO_2_ increase in the whole model domain (Figure 5A). Additionally, *g_0_* does not impact the modeled peak GRR (Figure 5A), the reason for which is discussed below. These results indicate that the optimal cell size of diatoms in response to the CO_2_ increase tends to be small when the environmental conditions favor their growth but can be larger when other factors are also limiting.

We also compared the sensitivity of the modeled peak GRR (Figure 5B) and optimal cell size (Figure 5C) to various model parameters. The CO_2_ supply rate, mainly determined by the production of the CO_2_ permeability (*P*) and CO_2_ gradient across the cytoplasmic membrane (△_*CO2*_) (Eqs. 3, 4), has a positive relationship with the optimal cell size but does not impact the GRR. This can be more clearly understood from a model experiment with a 50% higher *P* (Supplementary Figure 3A): The cell needs to use additional HCO_3_^–^ at large cell sizes with increased CO_2_ supply. Hence, the curves of HCO_3_^–^ acquisition energy under both HC and LC move toward larger cell sizes without noticeable changes in the magnitude of the energy cost, leading to a moderately elevated optimal cell size but unchanged peak GRR (Figures 5B,C; Supplementary Figure 3A). Similarly, a smaller maximal daily growth rate (*g_0_*) also leads to a larger optimal cell size but no effect on peak GRR (Figure 5 and Supplementary Figure 3B) because the lowered carbon demand also allows larger cells to only use CO_2_. In contrast, reduced CO_2_ leakage (*l_k_*) lowers the requirement of HCO_3_^–^ acquisition and shrinks the difference in energy cost between HC and LC, causing a decreased GRR and enlarged optimal cell size (Figure 5 and Supplementary Figure 3C).

The elevated energy cost rate of HCO_3_^–^ acquisition (*e*_*bc*_) substantially increases the peak GRR but has little effect on the optimal cell size (Figures 5B,C) because it mainly determines the energy allocation and not the cell size at which HCO_3_^–^ acquisition is needed. The high bulk-water CO_2_ concentration of HC (*HC*[CO_2_]_bulk_) greatly increases both the peak GRR and the optimal cell size (Figures 5B,C), which is expected because the cell under HC benefits more from higher CO_2_ while starting to use HCO_3_^–^ at a larger size. The minimum HCO_3_^–^ uptake proportion ($f_{m\,i\,n}^{b\,c}$) slightly impacts both the peak GRR and the optimal cell size: a higher $f_{m\,i\,n}^{b\,c}$ leads to a reduced model sensitivity to the change in CO_2_ and results in a lower peak GRR and a larger optimal cell size.

Our model sensitivity tests reveal that the peak GRR is largely determined by the magnitude of the seawater CO_2_ increase and the energy consumption rate in HCO_3_^–^ acquisition. Meanwhile, the optimal cell diameter increases with increasing CO_2_ supply (higher membrane permeability to CO_2_ and larger increase in seawater CO_2_) but decreases with cellular carbon demand, such as higher growth and leakage rates.

## Discussion

### Unimodal Relationship Between Growth Rate Response and Cell Size

Our theoretical model reveals a unimodal relationship between the GRR and diatom cell size. Although the increased CO_2_ concentration and acidified seawater can have multiple physiological effects on diatoms, our model considers the most direct effect: the variations in species and rates of inorganic carbon acquired by diatoms. The absolute rates of growth do not solely determine the competition among phytoplankton because they can also be controlled by other factors, such as zooplankton grazing and viral lysis, which is also suggested by the fact that phytoplankton with different growth rates coexist in the ocean. A phytoplankton species with a stronger enhancement in the growth rate from the CO_2_ increase, can have a better advantage and reach higher biomass when competing with other phytoplankton, which is also confirmed by a modeling study (Dutkiewicz et al., 2015). Therefore, the unimodal relationship between GRR and diatom cell size shown by our model suggests a competitive advantage for middle-sized diatoms of ∼7 μm in the future high-CO_2_ ocean.

It is generally true that organisms limited more strongly by a resource should benefit more from its repletion, but only if there are no substitute resources. CO_2_, for example, is not the only carbon resource for phytoplankton and can be complemented by, although not preferentially, HCO_3_^–^ when CO_2_ is insufficient. This is the important reason why our model reveals that middle-sized diatoms can have a competitive advantage over larger diatoms from elevated CO_2_, even though the latter is limited more strongly by CO_2_.

**FIGURE 6:**
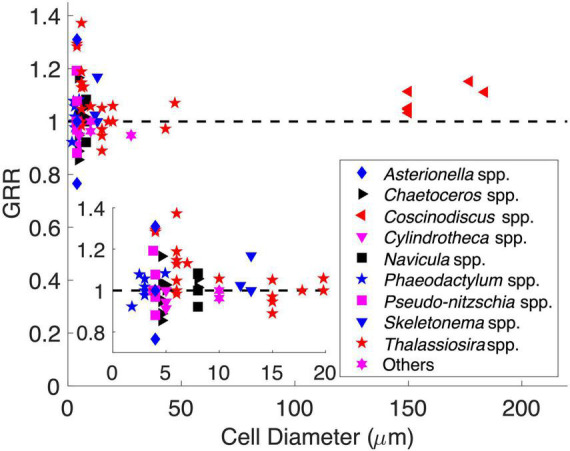
Historical data of diatom CO_2_ enrichment experiments showing the growth rate response (GRR) over cell diameter. The dashed line represents no response (GRR = 1). The inset is magnified to a cell range of 1–20 μm.

We also explored the GRR–cell size relationship using historical data from CO_2_ manipulation experiments of diatoms. The originally reported GRR in those experiments was first interpolated to the same degree of CO_2_ enrichment (Supplementary Figure 4 and Supplementary Table 2, see section “Materials and Methods”). The adjusted GRRs varied greatly (0.7–1.4) and did not support the previous speculation that the GRR should increase with cell size (Spearman’s correlation, *p* = 0.59) (Figure 6). The GRRs also did not directly show the same pattern as that proposed by our model. However, all GRRs higher than 1.2 were only found in cell diameters of 3–7 μm, while the GRRs at other cell diameters were mostly lower than 1.1 (Figure 6). This may indicate that the growth rate of diatoms in this middle cell size range had the greatest potential to respond to increasing seawater CO_2_. Additionally, the GRRs in the same size range also varied the most (Figure 6), suggesting that the GRR of middle-sized diatoms can be impacted by different factors and mechanisms and/or be species specific. Interestingly, considering the species in the genus *Thalassiosira* that had the largest cell diameter range (3.9–47 μm) in our dataset, their GRRs decreased with cell diameter (Spearman’s correlation = −0.45, *p* < 0.05), with GRRs in cell diameter ≤ 7 μm (1.14 ± 0.13, mean ± s.d.; *n* = 11) significantly higher than those in cells > 7 μm in diameter (1.00 ± 0.06, mean ± s.d.; *n* = 10) (*t*-test, *p* < 0.01). Nevertheless, these historical data showed some consistency with our model, while the GRR can be a synergistic result impacted not only by cell size but also by other physiological and ecological characteristics and culturing conditions. More studies are needed to reveal complex mechanisms controlling the GRR.

Other studies may directly or indirectly support our model. Hancock et al. (2018) cultured a near-shore Antarctic community over a gradient of CO_2_ levels, showing that the abundance of nanosized diatoms (2–20 μm in diameter) increased with CO_2_, while larger microplanktonic diatoms (>20 μm in diameter) and smaller discoid centric diatoms of 1–2 μm in diameter had no significant response to CO_2_. Other studies also showed that middle-sized diatom species (∼8 μm) had an increased contribution to the community under increased CO_2_ (Hoppe et al., 2013; Eggers et al., 2014).

We also constructed a new scheme of the maximal seawater CO_2_ supply rate to diatom cells as a function of cell size. Compared to other studies (Milligan et al., 2009; Reinfelder, 2011), the new scheme solves a cell size–dependent CO_2_ concentration near the cell surface (Supplementary Figure 1). Our scheme introduces a new constant parameter, the CO_2_ permeability of the cell membrane. Although elevated seawater CO_2_ can lead to a saturated CO_2_ supply and reduce the effective CO_2_ permeability (e.g., Sultemeyer and Rinast, 1996), our scheme is constructed to study the maximal effect of seawater CO_2_ increase and therefore uses the maximal potential of CO_2_ permeability, which theoretically is determined by the number of channels such as pores and aquaporins (Matsui et al., 2018; Blanco-Ameijeiras et al., 2020) allowed per unit area of the cell membrane and should be independent of cell size. Limited data on diatom CO_2_ permeability (Hopkinson et al., 2011) also do not show a clear relationship to cell size. Our model scheme therefore can be more suitable to quantify the relationship between phytoplankton cell size and their maximal potential for CO_2_ uptake.

### Model Experiments of Additional Processes

The growth rates of diatoms are generally inversely related to cell sizes (Cosper, 1982; Sarthou et al., 2005; Marañón, 2015), which could result from a lower CO_2_ supply rate to larger diatoms, as observed in this study, and/or other size-dependent physiological characteristics of diatoms. This implies that the constant model parameter of maximal growth rate *g_0_* can in reality decline with increasing cell size. We then conducted a model experiment by using an empirical function for diatoms (Sarthou et al., 2005):


(14) $${\text{g}}_{0}=3.4\,{\text{V}}^{-0.13}$$

where *V* was the cell volume (μm^3^). In the results, the unimodal pattern between GRR and cell size remained (Supplementary Figure 5), although the optimal cell size increased to 45 μm, which was expected because of the negative relationship between the optimal cell size and *g_0_* (Figure 5B).

As eCA can be potentially important in particularly large phytoplankton in converting HCO_3_^–^ to CO_2_ extracellularly (Martin and Tortell, 2008; Tortell et al., 2008; Chrachri et al., 2018) and therefore saving energy on HCO_3_^–^ acquisition, we tested its role by increasing the conversion rate of HCO_3_^–^ to CO_2_ in the model by an enhancement factor (*f*_*eCA*_) of 2–10 (Tortell et al., 2008). As the ratio of the chemical conversion between HCO_3_^–^ and CO_2_ to the CO_2_ diffusivity is $R\,\sqrt{k^{\prime }/D}$ (Eq. 2) (Reinfelder, 2011), the CO_2_ supply (*F_T_*) was then enhanced to a new rate:


(15) $${\text{F}}_{T}^{{}^{\prime }}=\frac{1+{\text{f}}_{\mathrm{eCA}}\,\text{R}\,\sqrt{{\text{k}}^{{}^{\prime }}/\text{D}}}{1+\text{R}\,\sqrt{{\text{k}}^{{}^{\prime }}/\text{D}}}\,{\text{F}}_{T}$$

In the model experiments, although the GRR was elevated in the large cells, the unimodal pattern between the GRR and cell size, the optimal cell diameter, and the magnitude of peak GRR were barely changed (Supplementary Figure 6A). We also conducted another model experiment by linearly increasing *f*_*eCA*_ from 1 (i.e., no enhancement) at the smallest cell diameter (2 μm) to 10 at a cell diameter of 200 μm to mimic a hypothetical scenario of stronger eCA of larger cells, and still obtained a similar pattern between the GRR and cell size, except that the GRR remained at a constant low level when the cell diameter was >100 μm (Supplementary Figure 6B).

The two parameters *D* and *k*′ used in the CO_2_ reaction–diffusion kinetics (Eq. 2) are fixed in our model, while in reality, they change with temperature (Wolf-Gladrow and Riebesell, 1997). A sensitivity test of temperature-dependent *D* and *k*′, however, showed no obvious effect on the model results (Supplementary Figure 7).

The above experiments can partly support the robustness of our model results, suggesting that the negative correlation between diatom growth rates and cell size, the extracellular conversion of HCO_3_^–^ to CO_2_, and temperature do not change the unimodal relationship between the GRR and the cell size of diatoms.

### Global Prediction

**FIGURE 7:**
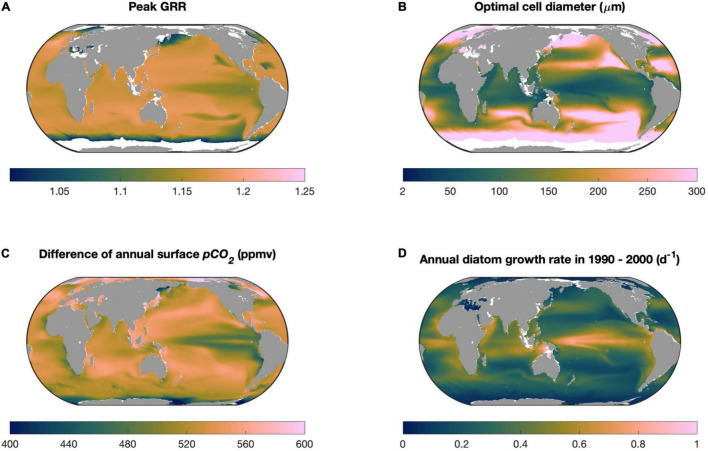
Model projections in the global ocean. **(A)** Modeled peak growth rate response (GRR) and **(B)** optimal cell size within this century under the CESM-predicted RCP8.5 scenario, including **(C)** the increase in partial pressure of CO_2_ and **(D)** annual average diatom growth rate. White areas in **(A,B)** represent the locations where the modeled GRR is very weak (<1.01).

Our model sensitivity experiments reveal that the modeled optimal cell size enlarges with stronger seawater CO_2_ supplies associated with its higher concentration and permeability across the cell membrane, but diminishes with elevating cellular carbon demand determined by higher rates of cell growth potential (Figure 5). Among these factors, the magnitude of future CO_2_ increase and the maximal growth rate of diatoms can vary substantially in the global ocean. Particularly, as already discussed, our model does not represent the effects of other factors that can limit diatom growth. We then ran our model in the global ocean using the projected levels of these two variables in this century under the RCP8.5 scenario from an Earth system model (see section “Materials and Methods”), in which multiple limiting factors on diatom growth were simulated. The spatial variation in the modeled peak GRR was generally small in the global ocean, with the lowest value in the tropical Pacific upwelling zone (Figure 7A) associated with its smallest magnitude of CO_2_ increase (Figure 7C). The modeled optimal cell size, however, varied greatly from < 20 μm in tropical oceans to the highest modeled cell diameter (i.e., GRR increases monotonically with cell size) in the subpolar regions (Figure 7B). The modeled optimal cell size was systematically smaller than what was predicted in our theoretical model (Figures 2, 5B) because the CESM-simulated diatom growth rates incorporated other limiting factors and were mostly below 1.0/day (Figure 7D), much lower than those found in laboratories with ideal culturing conditions (Sarthou et al., 2005). The lowest optimal cell size was also in the tropical Pacific upwelling zone (Figure 7B), contributed synergistically by the high growth rates of diatoms and low magnitude of CO_2_ increase (Figures 7C,D).

Our projection for diatoms in the global ocean should be regarded at most as the first-order estimation. However, some interesting implications emerged from the projection. Diatoms are one of the important contributors to carbon export to the deep ocean (Falkowski et al., 2004), particularly in tropical oceans (Siegel et al., 2014, 2016). The relatively small optimal cell size projected in these regions (Figure 7B) implies that, without considering other effects, the size structure of the diatom community would not shift to larger species in the future high-CO_2_ ocean, which consequently would not lead to an increase in the sinking speed of organic particles and carbon export.

### Model Limitations

Carbonic anhydrase in diatoms can convert CO_2_ to HCO_3_^–^ in the cytoplasm and result in much-increased HCO_3_^–^ transport further into chloroplasts, as found for diatom *Phaeodactylum tricornutum* (Hopkinson et al., 2011; Hopkinson, 2014). Additionally, a high leakage of CO_2_ from the chloroplast to the cytoplasm has been suggested (Hopkinson et al., 2011), although it is unclear how much of the leaked CO_2_ further leaks to the environment or is converted to HCO_3_^–^ in the cytoplasm and transported back to the chloroplast. The CO_2_ leakage from the cell to the environment was already counted in the model. However, if there is substantial recycling of the leaked CO_2_ to HCO_3_^–^ in the cytoplasm, the rate of HCO_3_^–^ transfer across the chloroplast membrane can be further elevated. In other words, *e*_*bc*_ can possibly be higher for at least some diatoms, leading to higher GRRs (Figure 5A) but small changes in the optimal cell size (Figure 5B).

There are some limitations in our model that can be investigated in future studies. First, our model does not include the negative effects of the lower pH concomitant with elevated CO_2_, which can be important to some species or functional groups of phytoplankton (e.g., Taylor et al., 2012; McMinn et al., 2014; Cyronak et al., 2016; Hong et al., 2017; Gafar et al., 2018; Luo et al., 2019; Shi et al., 2019). If there is no relationship between the negative effects of lower pH and diatom cell sizes, which, however, is unclear, our modeled pattern between GRR and diatom cell sizes can still hold.

Second, RuBisCO in different diatoms shows greater variation in the Michaelis constant for CO_2_ (23–68 μM) (Young et al., 2016), indicating diverse patterns of CCM, RuBisCO kinetics, and catalytic features in diatoms. Our model does not separate diatom species but focuses on the general response of diatoms of different sizes to seawater CO_2_ increases. Nevertheless, the variation of these factors can change model parameters of the cell membrane permeability to CO_2_ (*P*) and the energy cost rate in HCO_3_^–^ uptake (*e*_*bc*_), among which a ± 50% change in the former can vary the optimal cell size in a range of 2–13 μm while that in the latter has no substantial effect (Figure 5C). That is, if the CO_2_ permeability is substantially lower than that used in our model, the GRR can be highest in the smallest diatoms and then decreases with increasing cell size.

Third, light appears to interact with elevated CO_2_ in determining the collective effect in cell size–dependent responses of diatoms, such as that we already discussed for the stronger response of larger diatoms to CO_2_ increase under very high light. Another study also proposed a light-based maximal benefit of CCM in middle-sized phytoplankton: The depletion of light near the center of large cells depresses photosynthesis and reduces the energy available for CCM, making CCM economically less efficient in large cells (Wirtz, 2011). This effect and our proposed mechanism, if valid, can synergistically intensify the competitive advantage of middle-sized diatoms under the CO_2_ increase.

Last, cultured diatoms also often respond differently to CO_2_ increases depending on the availability of nutrients such as nitrogen (Li et al., 2012), phosphorus (Sun et al., 2011), and silicon (Tatters et al., 2012), while these nutrients are not simulated in our model. Instead, our model experiments implicitly represent the limitation of these nutrients by applying a lower model parameter *g_0_*, showing that middle-sized diatoms are more likely to have better competition from the CO_2_ increase when these nutrients are not severely limiting (Figures 5, 7). Nevertheless, our model does not simulate the cell size–dependent limitation of nutrients on diatoms. In the future warmer and more stratified oceans, nutrients can become more limiting, and smaller diatoms may gain another competitive advantage because of their lower surface-to-volume ratios. Considering this effect, the increasing trend of GRR from small to middle-sized diatoms can be weakened or even reversed. However, the decreasing trend of GRR from middle-sized to large-sized diatoms may be even stronger.

Overall, despite the limitations of our model, the discussions above suggest that the GRR in the future high-CO_2_ ocean, in general, likely decreases from middle-sized to large-sized diatoms, except in the regions where the light is substantially high or nutrients are substantially limiting on diatoms. The GRR may also increase from small to middle-sized diatoms unless the cell membrane permeability to CO_2_ is low or nutrients become severely limiting, particularly on middle-sized diatoms.

In summary, this study constructed a theoretical model and revealed a competitive advantage of middle-sized diatoms of ∼7 μm over both small and large diatoms when seawater CO_2_ increases. Although the model is mostly based only on the basic principles of the carbonate equilibrium system and inorganic carbon diffusion with constraints on cellular energy allocations, it appears to be a robust pattern, although the exact cell size of which diatoms benefit most from the CO_2_ increase can change substantially. If the mechanism proposed in this study dominates, the diatom community in the future high-CO_2_ ocean may not shift toward larger cells, and carbon export by sinking diatoms may not be substantially stimulated, while we admit that other known and unknown factors can also influence the cell size–dependent responses of marine diatoms to the CO_2_ increase. Our simplified physiological model processes, or even more simply the size-dependent pattern revealed by our model, can be integrated into marine ecosystem models to improve the predictions of the size composition of diatoms, phytoplankton community structure, and productivity for future high-CO_2_ oceans.

## Data Availability Statement

The original contributions presented in the study are included in the article/Supplementary Material, further inquiries can be directed to the corresponding author.

## Author Contributions

Y-WL originated the concept for the study. Y-WL and QZ designed the numerical model, analyzed the results, and improved the numerical model. QZ coded the initial version of the model and performed numerical modeling. Both authors contributed to writing the manuscript.

## Conflict of Interest

The authors declare that the research was conducted in the absence of any commercial or financial relationships that could be construed as a potential conflict of interest.

## Publisher’s Note

All claims expressed in this article are solely those of the authors and do not necessarily represent those of their affiliated organizations, or those of the publisher, the editors and the reviewers. Any product that may be evaluated in this article, or claim that may be made by its manufacturer, is not guaranteed or endorsed by the publisher.

## Footnotes

1
https://www.earthsystemgrid.org/dataset/ucar.cgd.ccsm4.CESM_CAM5_BGC_LE.ocn.proc.monthly_ave.html
