# Supplementary material for: A Competitive Advantage of Middle-Sized Diatoms From Increasing Seawater CO2
Source: Front Microbiol. 2022 May 18;13:838629. doi: 10.3389/fmicb.2022.838629 (PMC9158336; doi:10.3389/fmicb.2022.838629)
Supplement: Supplementary file 1 [file Table_1.PDF]

Supplementary Table S1. Model variables and parameters.

Symbol	Default Value	Unit	Description	References
			Input variables	
R		μm	Cell radius	
$[CO_2]_{bulk}$		μM	CO_2 concentration in bulk water	
			Parameters	
D	1450	$\mu m^2\ s^{-1}$	Diffusion coefficient	(Wolf-Gladrow and Riebesell, 1997)
k'	0.018	s^{-1}	$k' = k_1 + k_4[OH^-]$; k_1 and k_4 is the rate constant for the hydration of CO_2 by reaction with H_2O and OH^-	(Wolf-Gladrow and Riebesell, 1997; Reinfelder, 2011)
e_{bc}	1.5	ATP $(mol\ C)^{-1}$	Energy cost rate of HCO_3^- uptake	(Liu et al., 2017)
e_g	3.5	ATP $(mol\ C)^{-1}$	Energy cost rate of cell growth	(Raven, 1991; Eichner et al., 2014)
g_0	3.0	d^{-1}	Maximal daily growth rate	(Sarthou et al., 2005)
$[CO_2]_{bulk}^{ref}$	10	μM	Reference bulk-water CO_2 concentration	
l_k	30%		CO_2 leakage as fraction of total carbon acquisition	This study and (Burkhardt et al., 2001; Raven and Beardall, 2016)
P	600	$\mu m\ s^{-1}$	CO_2 permeability of cell membrane	This study and (Hopkinson et al., 2011)
Δ_{CO2}	10%		Relative reduction of CO_2 concentration from near cell surface to cytoplasm	(Hopkinson et al., 2016)
f_{min}^{bc}	20%		Minimal HCO_3^- acquisition as a fraction of total carbon demand	(Burkhardt et al., 2001)
