# Supplementary material for: A Competitive Advantage of Middle-Sized Diatoms From Increasing Seawater CO2
Source: Front Microbiol. 2022 May 18;13:838629. doi: 10.3389/fmicb.2022.838629 (PMC9158336; doi:10.3389/fmicb.2022.838629)
Supplement: Supplementary file 2 [file Presentation_1.pdf]

Supplementary Material

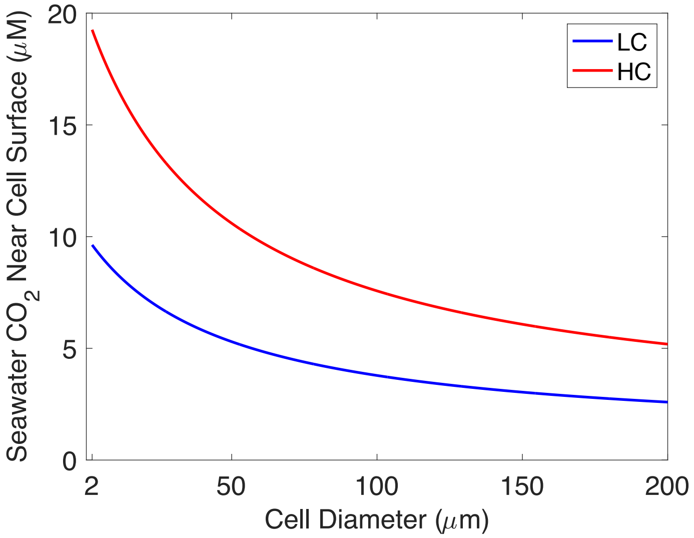

Supplementary Figure 1. Modeled CO_2 concentration at the cell surface as a function of cell diameter under low-CO_2 (LC) (blue) and high-CO_2 (HC) (red) conditions.

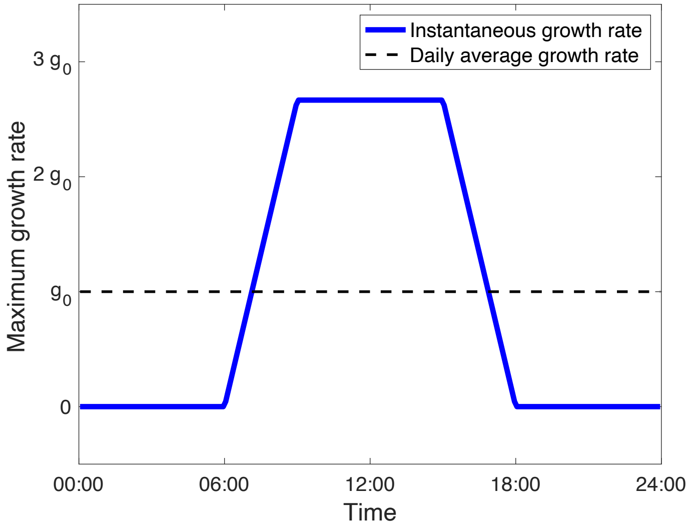

Supplementary Figure 2. Diel cycle of instantaneous maximal growth rate (blue line) of model phytoplankton cells as a result of changing light. The black dotted line represents the daily average maximal growth rate set by the model parameter g_0.

(A)

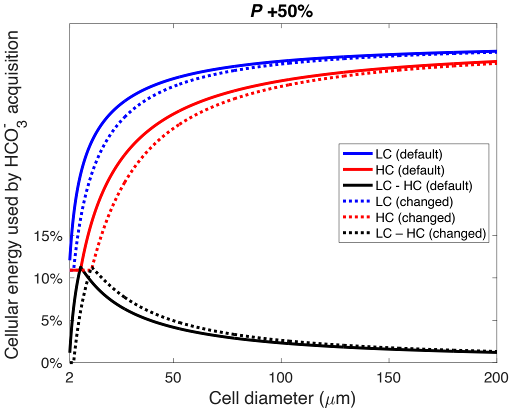

(B)

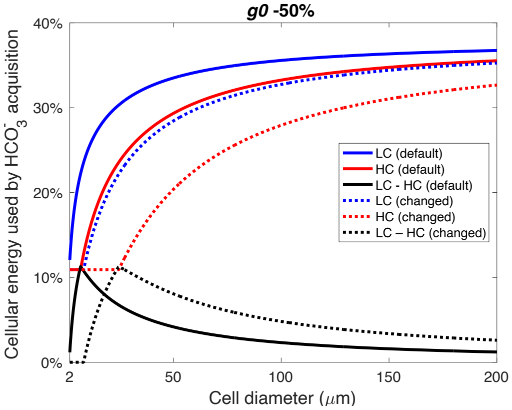

(C)

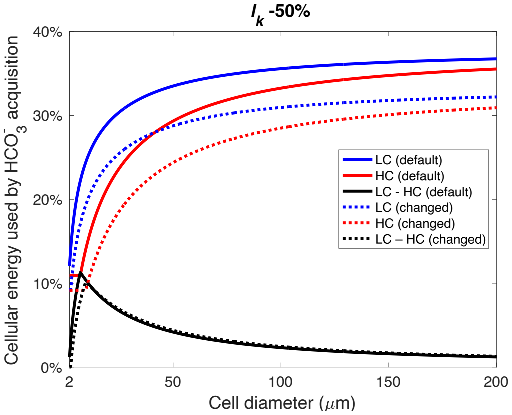

Supplementary Figure 3. The response of the modeled energy consumption for HCO_3^- acquisition to parameter changes as function of cell diameter. (**A**) Cell membrane permeability to CO_2 (P) increases by 50%, and (**B**) CO_2 leakage fraction (l_k) reduces by 50%. The energy for HCO_3^- acquisition is shown as the fraction of total cellular energy under the low-CO_2 (LC) (blue) and high-CO_2 (HC) (red) conditions, as well as the saved energy in HCO_3^- acquisition when the condition changed from LC to HC (black). The solid and dashed lines represent model results under the default and changed parameter values, respectively.

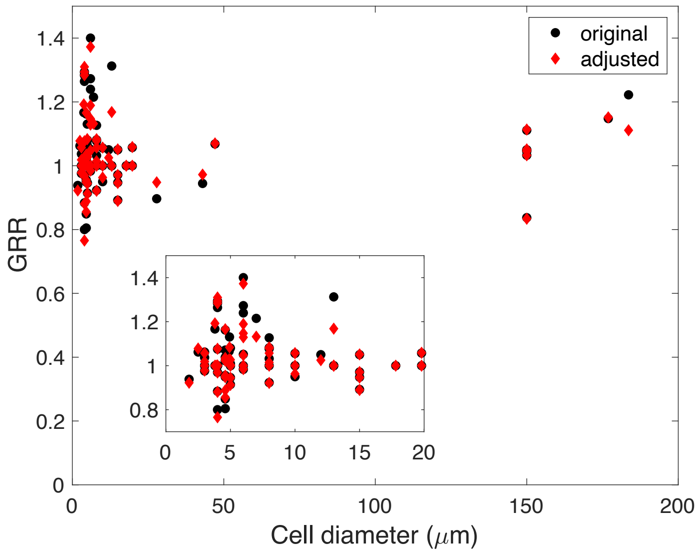

Supplementary Figure 4. Original reported experiment GRR and the GRR adjusted to the same CO_2-enrichment factor of 2. The inset is zoomed in to a cell range of 1–20 µm.

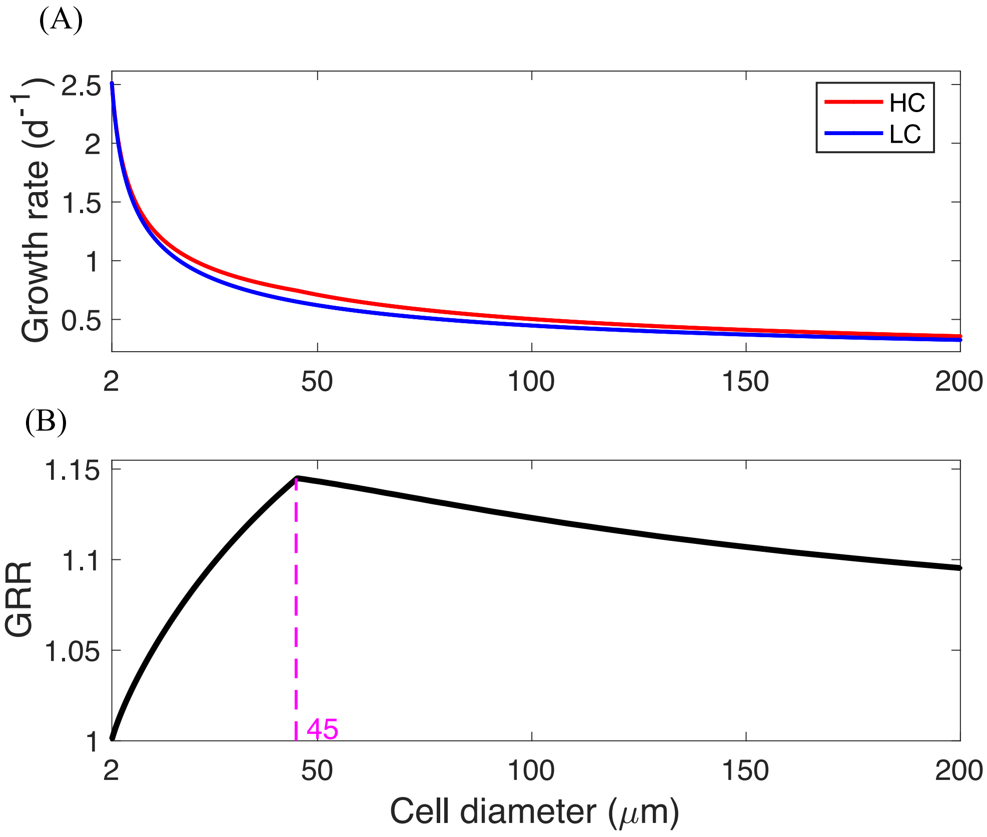

Supplementary Figure 5. Model experiments in which the daily maximal growth rate (g_0) decreases with cell size. (**A**) Modeled growth rate under the low-CO_2 (LC) (blue) and high-CO_2 (HC) (red) conditions, and (**B**) the corresponding growth rate response (GRR). The magenta dashed line and number in (**B**) represent the optimal cell diameter at peak GRR.

(A)

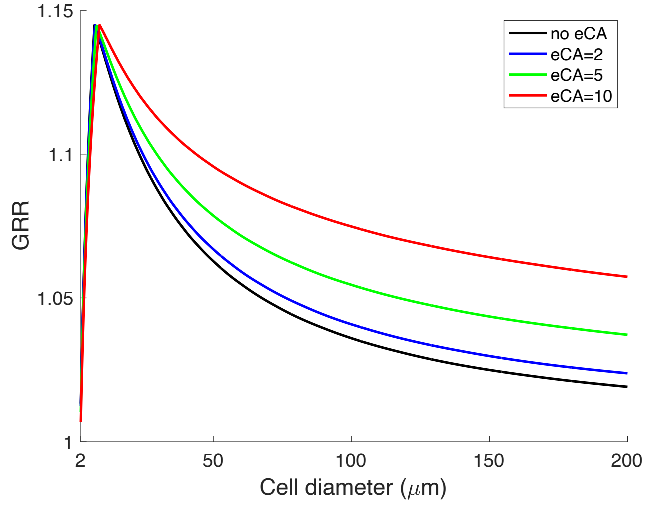

(B)

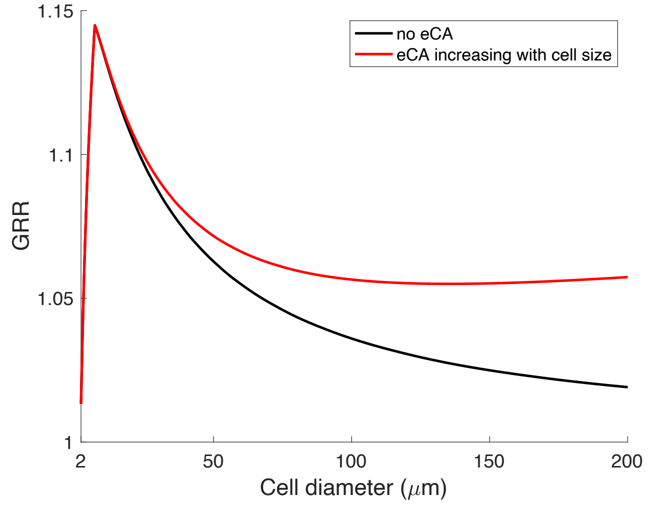

Supplementary Figure 6. Model experiments of extracellular carbonic anhydrase (eCA). (**A**) The modeled GRR with different eCA enhancement factors; note that the GRR lines approximately overlap when the cell size is smaller than 10 μm. (**B**) The comparison of GRR between the standard model without eCA (black line) and the experiment (red line) using a linearly increasing eCA with cell size (see texts).

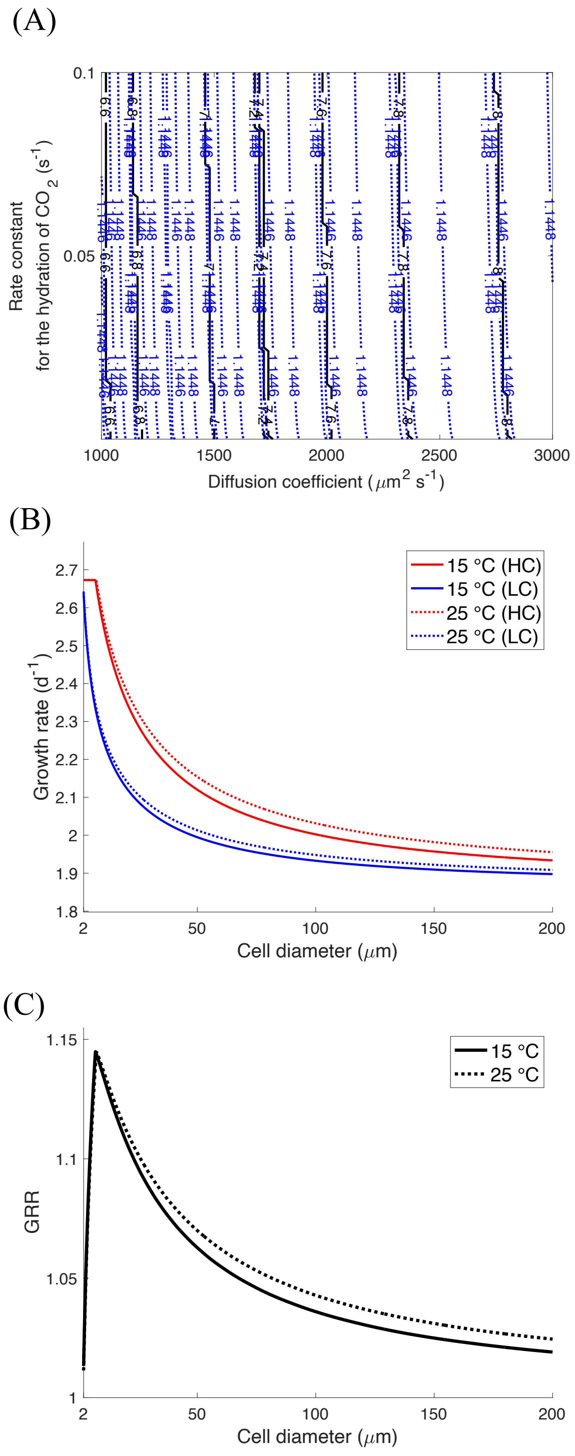

Supplementary Figure 7. Impact of diffusion coefficient (D) and combined rate constant (k') on the model. (**A**) Model sensitivity tests of peak GRR (black) and optimal cell diameter (μm; blue) as a function of parameters of D and k' (see main text for its definition). (**B**) Modeled growth rate and (**C**) GRR versus cell size at 15 °C (D = 1.45 × 10^3 μm^2 s^{-1}, k' = 0.018 s^{-1}, according to Wolf-Gladrow and Riebesell 1997) and 25 °C (D = 1.45 × 10^3 μm^2 s^{-1}, k' = 0.018 s^{-1}, according to Wolf-Gladrow and Riebesell 1997). Blue and red lines in (**B**) are for low-CO_2 (LC) and high-CO_2 (HC) conditions.
